# Trends in Transactional Sex Among Women at Risk for HIV in Rural Kenya During the First Year of the COVID-19 Pandemic

**DOI:** 10.1001/jamanetworkopen.2022.20981

**Published:** 2022-07-05

**Authors:** Aaron Richterman, Risper Bosire, Noora Marcus, Elizabeth F. Bair, Kawango Agot, Harsha Thirumurthy

**Affiliations:** 1 Division of Infectious Diseases, Hospital of the University of Pennsylvania, Philadelphia; 2 Impact Research and Development Organization, Kisumu, Kenya; 3 Department of Medical Ethics and Health Policy, University of Pennsylvania, Philadelphia

## Abstract

This cohort study examines trends in economic outcomes and behaviors associated with HIV transmission among women at risk in rural Kenya during the COVID-19 pandemic.

## Introduction

Transactional sex, or the exchange of sex for material support, is an important factor associated with the high risk of HIV acquisition among young women in sub-Saharan Africa.^1^ Because poverty is associated with women’s engagement in transactional sex, the economic consequences of the COVID-19 pandemic may be associated with increased HIV incidence. Several studies^2,3,4^ reported worsening economic conditions in low-income and middle-income countries during the initial months of the pandemic. Few studies have examined longer-term trends in economic outcomes or behaviors associated with HIV transmission.

## Methods

In this cohort study, we analyzed longitudinal data from cisgender women in Kenya who participated in a cluster randomized trial of an HIV self-testing intervention.^5^ Eligible women were HIV negative and reported 2 or more male sex partners in the past 4 weeks. Participants were enrolled between 2017 and 2018 and were administered surveys every 6 months during the trial, which ended in March 2020.^5^ We also conducted 2 telephone surveys between May and June 2020 and November 2020 and February 2021. Ethics committees at the University of Pennsylvania and Maseno University approved the study. Participants provided written informed consent. The study followed the Strengthening the Reporting of Observational Studies in Epidemiology (STROBE) reporting guideline.

We examined participants’ weekly income, employment hours, total number of sex partners and transactional sex partners in the past month, and prices associated with transactional sex. All monetary values are shown in US dollars. We compared early and later pandemic outcomes with those from the 6 months before the pandemic using regression models (linear except for Poisson for number of sex partners) with time fixed effects (to adjust for secular trends), individual fixed effects (to adjust for time invariant individual characteristics), cluster-robust SEs, and significance set at 2-sided *P* < .05. We used SAS statistical software version 9.4 (SAS Institute) and Stata statistical software version 17 (StataCorp). Data analysis was performed from March to June 2021.

## Results

A total of 2090 women were enrolled; 1725 (83%) completed the first telephone survey, and 1731 (83%) completed the second telephone survey during the pandemic. Participants had a median (IQR) age of 27 (23-32) years at the start of the pandemic, and 1980 (96%) reported having ever engaged in transactional sex at study enrollment. Income was stable during the prepandemic period but decreased significantly during the pandemic (Figure, panel A). Compared with a mean weekly income of $10.90 (95% CI, $9.70-$12.70) in the 6 months before the pandemic, income was lower in the early (mean, $5.60; 95% CI, $4.80-$6.60; *P* < .001) and later (mean, $9.40; 95% CI, $8.50-$10.90; *P* < .001) pandemic periods. Compared with the 6 months before the pandemic, participants worked 16 fewer hours per week (95% CI, 14-17 hours per week) during the early pandemic period and 10 fewer hours per week (95% CI, 8-12 hours per week) during the later pandemic period.

**Figure.**
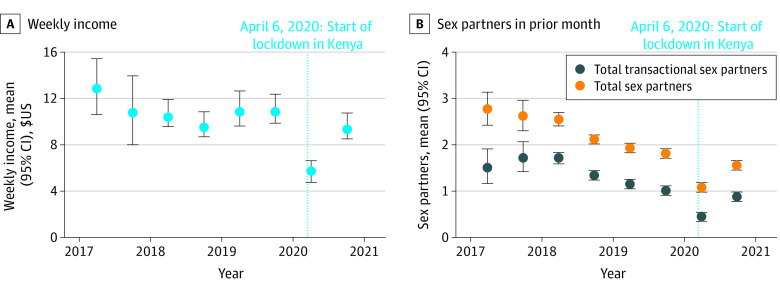
Trends in Participants’ Weekly Income and Number of Total and Transactional Sex Partners Over The Past Month Panel A shows trends in participants’ mean weekly income (with 95% CIs denoted by error bars). Panel B shows trends in participants’ mean number of total and transactional sex partners over the past month (with 95% CIs denoted by error bars). April 6, 2020, denotes the start of the initial lockdown period in Kenya, when mobility restrictions and business closures were put in place.

During the 6 months before the pandemic, participants reported a mean of 1.8 total sex partners (95% CI, 1.7-1.8 total sex partners) and 1.0 transactional sex partner (95% CI, 0.9-1.1 transactional sex partners) in the previous month. This decreased to a mean of 1.1 total partners (95% CI, 1.0-1.1 total partners; *P* < .001) and 0.5 transactional partner (95% CI, 0.4-0.5 transactional partner; *P* < .001) during the early pandemic period (Figure, panel B). Participants reported a mean of 1.5 total partners (95% CI, 1.5-1.6 total partners) and 0.9 transactional partners (95% CI, 0.8-0.9 transactional partners) during the later period, approaching prepandemic levels but significantly lower (*P* < .001). The mean price of transactional sex per act with condoms increased from $8.00 (95% CI, $7.20-$9.10) during the 6 months before the pandemic to $10.20 (95% CI, $8.90-$12.00; *P* = .01) during the later pandemic period, and the mean price for sex without condoms increased from $10.40 (95% CI, $9.60-$11.80) to $12.40 (95% CI, $10.90-$14.50; *P* = .04).

## Discussion

In this cohort study, among Kenyan women with high risk of HIV exposure, the first year of the COVID-19 pandemic was associated with a persistent decline in income and employment. Although the incidence of transactional sex decreased during the pandemic’s early phase, by the end of 2020 it approached prepandemic levels. Heightened poverty may be one reason for the resurgence in transactional sex, which is likely associated with increased risk of HIV acquisition.^6^ Study limitations include the lack of assessments after the first year of the pandemic. Intersecting vulnerabilities to poverty, HIV, and COVID-19 should be considered by pandemic policies in low-income settings.
